# Supplementary material for: Mad1 destabilizes p53 by preventing PML from sequestering MDM2
Source: Nat Commun. 2019 Apr 4;10:1540. doi: 10.1038/s41467-019-09471-9 (PMC6449396; doi:10.1038/s41467-019-09471-9)
Supplement: Supplementary file 1 — Supplementary Information [file 41467_2019_9471_MOESM1_ESM.pdf]

SUPPLEMENTARY INFORMATION

Mad1 destabilizes p53 by preventing PML from sequestering MDM2

Wan, J. et al.

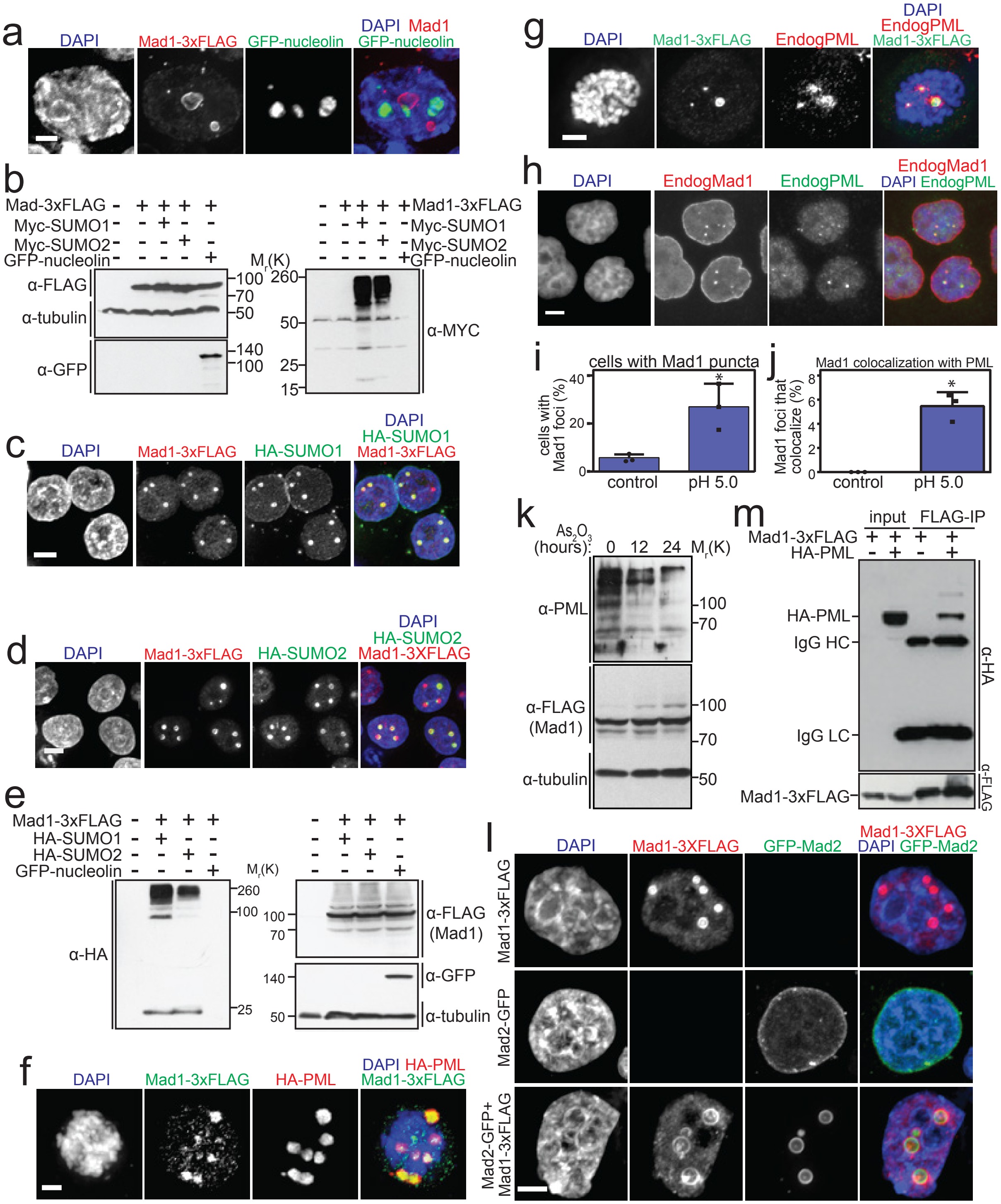

Supplementary Figure 1. Upregulated Mad1 localizes to PML nuclear bodies (NBs). (a) Upregulated Mad1 does not localize to nucleoli in the absence of DNA damage. MDA-MB-231 cells were co-transfected with constructs expressing Mad1-3xFLAG and GFP-nucleolin and analyzed by immunofluorescence with anti-Mad1 antibodies. Scale bar = 2.5 μm. (b) Equivalent protein loading in experiments showing colocalization of upregulated Mad1 with proteins localized to PML NBs but not nucleoli. Protein extracts from the cells in Figures 1a, 1b and

Supplementary figure 1a were analyzed using the indicated antibodies. (c-e) Upregulated Mad1 colocalizes with SUMO1 and SUMO2 in HeLa cells. HeLa cells were co-transfected with constructs expressing Mad1-3xFLAG and HA-SUMO1 (c) or HA-SUMO2 (d) and analyzed by immunofluorescence with anti-Mad1 and anti-HA antibodies. Scale bars = 5 µm. (e) Protein extracts from cells in Supplementary figure 1c-d were analyzed using the indicated antibodies. (f-g) Mad1 colocalizes with PML nuclear bodies in mitosis. Scale bars = 5 µm. (f) HeLa cells stably expressing Mad1-3xFLAG were transfected with a plasmid expressing HA-PML and analyzed by immunofluorescence with anti-Mad1 and anti-HA antibodies in prometaphase. (g) HeLa cell stably expressing Mad1-3xFLAG analyzed by immunofluorescence with anti-FLAG and anti-PML antibodies in prometaphase. (h-j) Under stressful growth conditions, a fraction of endogenous Mad1 localizes to PML nuclear bodies. (h) Under optimal growth conditions, nuclear puncta formed by endogenous Mad1 do not colocalize with PML. Scale bar = 5 µm. (i) Quantitation +/- SD showing that growth at pH 5 increases the percentage of cells with nuclear puncta of endogenous Mad1. n > 200 from each of 3 independent experiments. (j) Quantitation +/- SD showing a small fraction of endogenous Mad1 colocalizes with PML at pH 5. n > 100 puncta from each of 3 independent experiments. (k) PML but not Mad1 is degraded by treatment with As_2O_3. Protein extracts from HeLa cells treated with As_2O_3 for the indicated number of hours, as in Figure 1g-h, were immunoblotted with the indicated antibodies. (l) The localization of Mad2 into PML nuclear bodies is dependent on Mad1. HeLa cells were transfected with Mad2-GFP and/or Mad1-3xFLAG and analyzed by immunofluorescence using anti-FLAG antibodies and GFP fluorescence. Mad2 only forms nuclear puncta in the presence of upregulated Mad1. Scale bar = 5 µm. (m) Mad1 interacts with PML. 293T cells were transfected with Mad1-3xFLAG and HA-PML isoform IV and immunoprecipitated using beads coupled to anti-FLAG antibodies. Blot is representative of 3 independent experiments. *=$p<0.05$ by t test. For specific *P* values, see Source Data file.

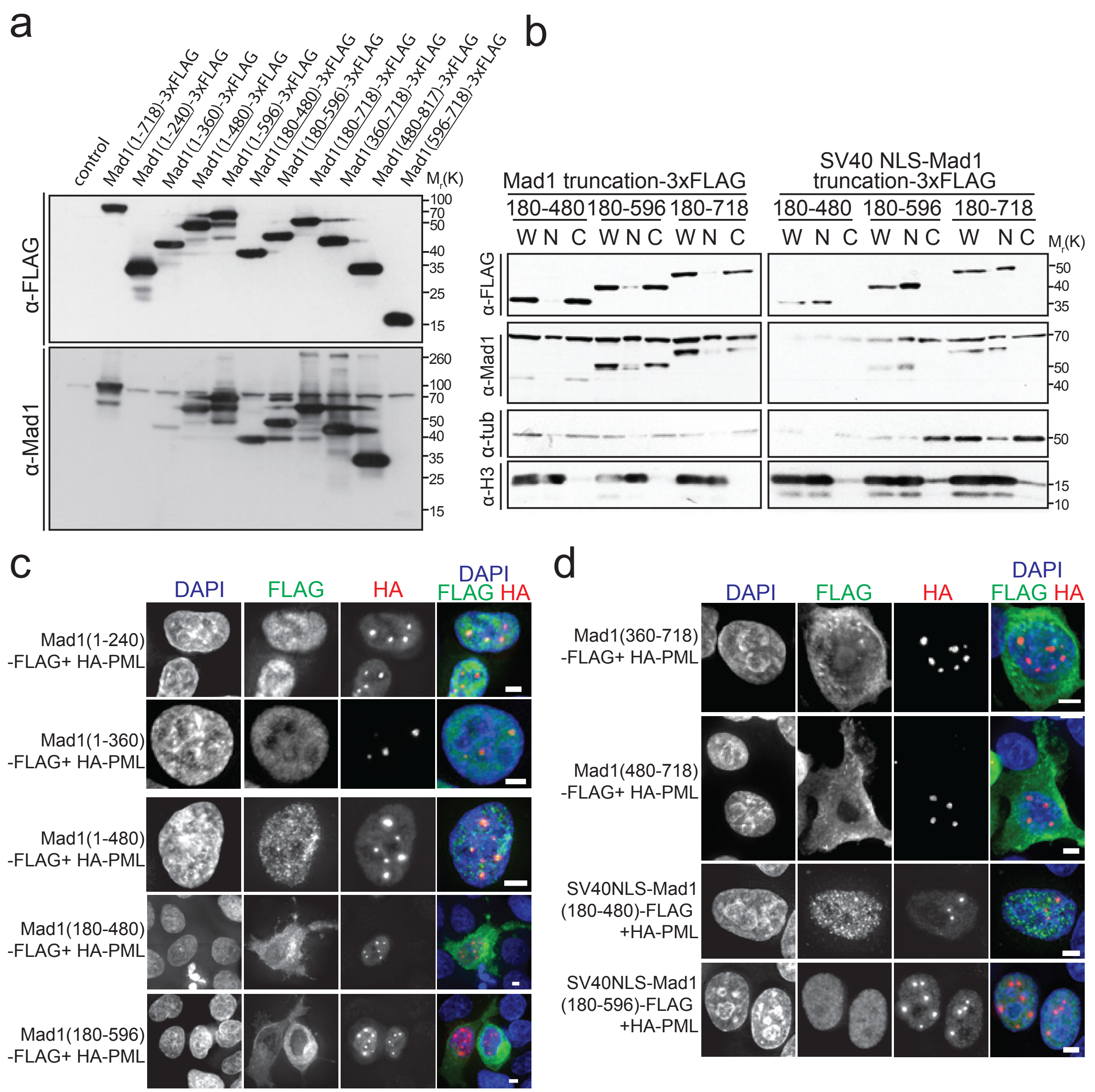

Supplementary Figure 2. The C-terminal domain of Mad1 and N-terminus of PML are necessary for their interaction. (a) Lysates of HeLa cells transfected with plasmids encoding various Mad1 fragments were blotted with anti-FLAG or Mad1 showing similar expression levels of the Mad1 truncations. Mad1 truncations were used for immunoprecipitation and co-immunoprecipitation experiments in Fig. 2b-c and localization experiments in Fig. 2d-f. (b) Fractionation of Mad1 and Mad1 fragments into whole cell lysate (W), nuclear (N) or cytoplasmic (C) fractions demonstrating that Mad1 fragments lacking the Mad1 nuclear import signal (NIS; aa 1-180) are cytoplasmic, but can be driven into the nuclear fraction by fusion with the SV40 NLS. HeLa cells transfected with plasmids expressing the indicated Mad1 fragments were fractionated and immunoblotted using anti-FLAG and Mad1 antibodies. Tubulin is used as a cytoplasmic marker and histone H3 as a nuclear marker. (c-d) HeLa cells co-transfected with plasmids encoding the indicated Mad1 fragment and HA-PML (isoform IV) were analyzed by

immunofluorescence with anti-Mad1 and anti-HA antibodies. (c) Mad1 fragments containing the NIS but lacking the CTD are nuclear but do not accumulate in PML NBs. Fragments lacking the NIS but containing the NES (aa 181-274) are predominantly cytoplasmic. (d) Fragments lacking both the NIS and NES (aa 1-274) are predominantly cytoplasmic. Fusion of fragments lacking the NIS and the CTD to an SV40 NLS is sufficient to drive the fragments into the nucleus, but insufficient for colocalization with PML.

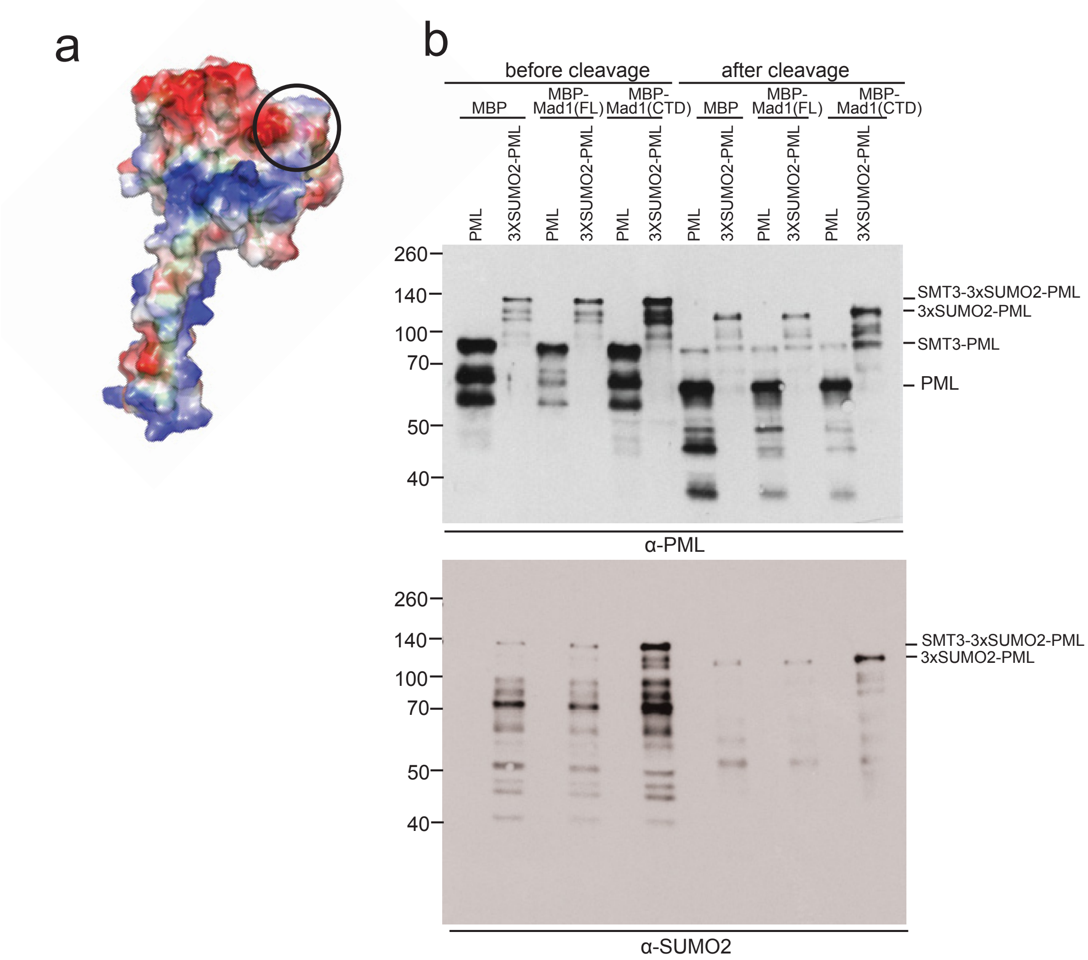

Supplementary Figure 3. Sumoylation promotes the direct binding of the Mad1 CTD to PML. (a) Swiss model predicted structure of human Mad1 CTD domain, SMTL id: 4dzo.1.A. Color key for amino acid side chain types at the protein surface: white = hydrophobic; red = negative; blue= positive; pink = polar neutral; yellow = aromatic; green = proline. The 689 leucine and 690 isoleucine are circled. (b) Input of PML and 3xSUMO2-PML for Figure 3f. The tag used to increase solubility (yeast SUMO SMT3) was successfully cleaved, as shown by removal of the higher molecular weight band.

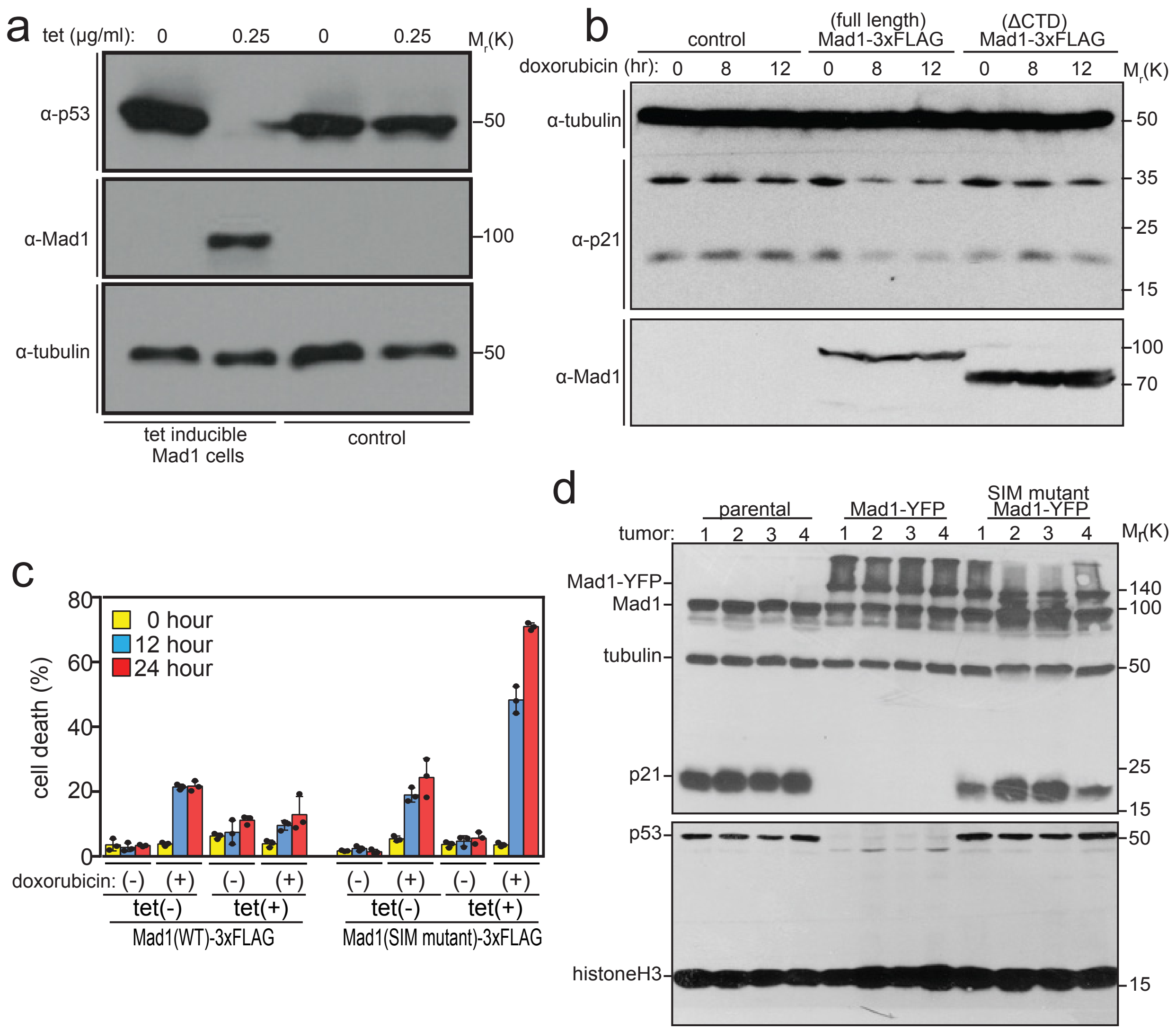

Supplementary Figure 4. Destabilization of p53 by Mad1 requires PML binding. (a) Tet-inducible expression of Mad1 in DLD1 cells prevents stabilization of p53 in response to DNA damage caused by the topoisomerase II inhibitor doxorubicin. DLD1 cells expressing wild type Mad1 in a tet-inducible manner were treated with 0.25 µg/mL tet for 24 hours and then with doxorubicin (4 µg/mL) for 12 hours. (b) Upregulation of Mad1 prevents p21 accumulation in response to DNA damage. HeLa cells stably expressing full length Mad1 or Mad1-ΔCTD in a tet-inducible manner were treated with tet for 24 hours and then treated with doxorubicin (2 µg/mL) for the indicated times. Parental HeLa cells which express the tet repressor but not tet-inducible Mad1 were used as a control. (c) Upregulation of wild type but not SIM mutant Mad1 reduces cell death as a consequence of DNA damage. HeLa cells were tet treated for 24 hours before addition of 2 µg/mL doxorubicin. Cell death (+/- SD) was scored using trypan blue assay. n > 200 cells from each of three independent experiments. (d) MDA-MB-231 orthotopic tumors expressing Mad1-YFP contain lower levels of p53 and its effector p21 than parental MDA-MB-231 tumors. Mutation of the Mad1 SIM, which is necessary for interaction with PML, rescues expression of p53 and p21. Protein lysates from tumors collected at day 40 post-injection were analyzed by immunoblot.

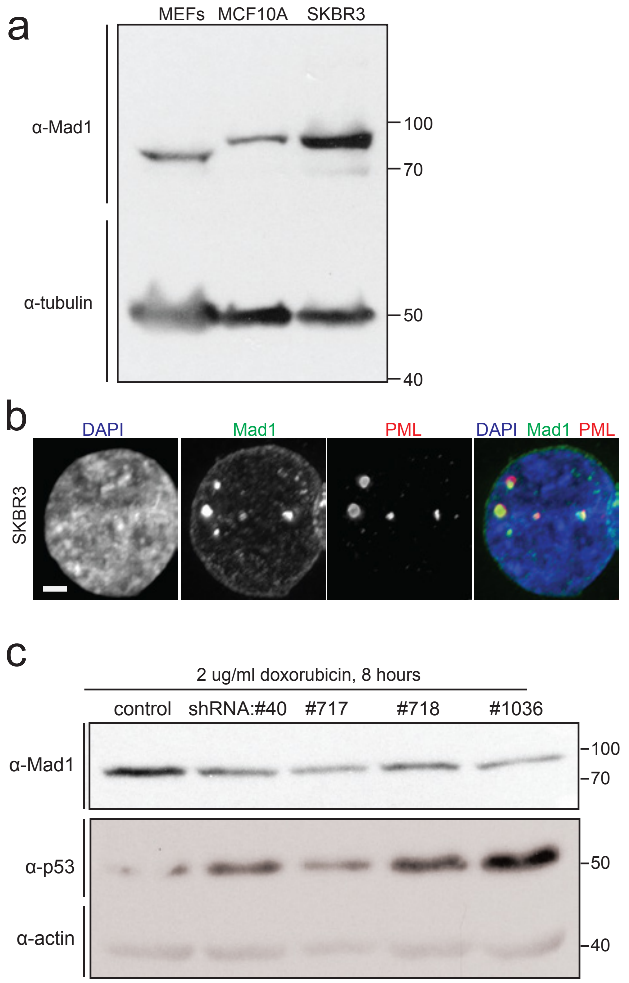

Supplementary Figure 5. Elevated levels of endogenous Mad1 regulate p53 accumulation in breast cancer cells. (a) SKBR3 cells highly express Mad1 without experimental manipulation. MEFs = mouse embryonic fibroblasts. α-tubulin is shown as a loading control. (b) Endogenous Mad1 colocalizes with endogenous PML in SKBR3 cells. Scale bar, 2.5 µm. (c) Endogenous Mad1 destabilizes p53 in SKBR3 cells. Partial depletion of Mad1 in SKBR3 cells stably expressing 4 distinct shRNA sequences results in increased protein levels of p53 after DNA damage caused by doxorubicin. Actin is shown as a loading control.

Supplementary Table 1: *List of vectors and primers used in this study*

Vector	Primer name/sequence (forward)		Primer name/sequence (reverse)	
pcDNA5/FRT/TO-3xFLAG	Xho I-3Flag-Sense	TCGAG ATG GAC TAC AAA GAC CAT GAC GGT GAT TAT AAA GAT CAT GAT ATC GAT TAC AAG GAT GAC GAT GAC AAG GGGCC	3xFlag-Apa1-Anti	CCTTGTCATCGTCATCCTTGTAATCGATATCATGATCTTTATAATCACCGTCATGGTCTTTGTAGTCCATC
pcDNA5/FRT/TO-Mad1-3xFLAG	BamH1-Mad1-sense	CCTGGATCCACCATGGAAGACCTG	EcoR V-Mad1-Anti	TTCTAGATATCTCGTCGCCACGGTCTG
pcDNA5/FRT/TO-Mad1(SIM mutant)-3xFLAG	Mad1-SIM-mutant-Sense	CTGCGTCGACAAGACAGCATCCCTGCCTT	Mad1-SIM-mutant-anti	ATGAAACTCTTTTTTTTCGCCCACGGTGTGTGA
pcDNA5/FRT/TO-Mad1(1-240)-3xFLAG	BamH1-Mad1-sense	CCTGGATCCACCATGGAAGACCTG	Mad1-240-anti-EcoRV	CCTCTGATATCTCGCTGCATCCTGC
pcDNA5/FRT/TO-Mad1(1-360)-3xFLAG	BamH1-Mad1-sense	CCTGGATCCACCATGGAAGACCTG	Mad1-360-anti-EcoRV	TATTCGATATCCCGCGCTGTTCTTG
pcDNA5/FRT/TO-Mad1(1-480)-3xFLAG	BamH1-Mad1-sense	CCTGGATCCACCATGGAAGACCTG	Mad1-480-anti-EcoRV	GGGGAGATATCCTGCTCCATCTCCA
pcDNA5/FRT/TO-Mad1(1-596)-3xFLAG	BamH1-Mad1-sense	CCTGGATCCACCATGGAAGACCTG	Mad1-596-anti-EcoRV	ATCCTGATATCATGGCAGACTCGCG
pcDNA5/FRT/TO-Mad1(180-480)-3xFLAG	BamH1-180-Mad1-sense	ATAGGATCCACAATGAAGCGCCTGG	Mad1-480-anti-EcoRV	GGGGAGATATCCTGCTCCATCTCCA
pcDNA5/FRT/TO-Mad1(180-596)-3xFLAG	BamH1-180-Mad1-sense	ATAGGATCCACAATGAAGCGCCTGG	Mad1-596-anti-EcoRV	ATCCTGATATCATGGCAGACTCGCG
pcDNA5/FRT/TO-Mad1(180-718)-3xFLAG	BamH1-180-Mad1-sense	ATAGGATCCACAATGAAGCGCCTGG	EcoR V-Mad1-Anti	TTCTAGATATCTCGTCGCCACGGTCTG
pcDNA5/FRT/TO-Mad1(360-718)-3xFLAG	BamH1-Mad1-360-Sense	TATGGATCCATAATGGGGCTGGAGAA	EcoR V-Mad1-Anti	TTCTAGATATCTCGTCGCCACGGTCTG
pcDNA5/FRT/TO-Mad1(480-718)-3xFLAG	BamH1-Mad1-481-Sense	CCGGGATCCACCATGAAGTCTCAGT	EcoR V-Mad1-Anti	TTCTAGATATCTCGTCGCCACGGTCTG
pcDNA5/FRT/TO-Mad1(597-718)-3xFLAG	BamH1-596-Sense	TAATGGATCCACTATGCCATCGTCCA	EcoR V-Mad1-Anti	TTCTAGATATCTCGTCGCCACGGTCTG
pcDNA5/FRT/TO-NLS-Mad1(180-480)-3xFLAG	BamH1-NLS-180-Mad1	TGAGGATCCATGCCACCAAAAAAAAAACGTAAAGTTATGAAGCGCCTGG	Mad1-480-anti-EcoRV	GGGGAGATATCCTGCTCCATCTCCA
pcDNA5/FRT/TO-NLS-Mad1(180-596)-3xFLAG	BamH1-NLS-180-Mad2	TGAGGATCCATGCCACCAAAAAAAAAACGTAAAGTTATGAAGCGCCTGG	Mad1-596-anti-EcoRV	ATCCTGATATCATGGCAGACTCGCG
pcDNA5/FRT/TO-NLS-Mad1(180-718)-3xFLAG	BamH1-NLS-180-Mad3	TGAGGATCCATGCCACCAAAAAAAAAACGTAAAGTTATGAAGCGCCTGG	EcoR V-Mad1-Anti	TTCTAGATATCTCGTCGCCACGGTCTG
pcDNA5/FRT/TO-Mad1(1-274+596-718)-3xFLAG	Mad1-596-O/L-274-FOR	CGGGAGATGAGAGAG TCGTCCAAGGAGGTG	Mad1-274-O/L-596-Rev	CACCTCCTTGGACGA CTCTCTCATCTCCCG

pcDNA5/FRT/TO-HA-PML(1-229)	BamH1-PML-Sense	CTATGGATCCATGGTCGAGCCTG	PML-229-EcoRV-antisense	GGTCGATATCTGTCGCACTTGAGCT
pcDNA5/FRT/TO-HA-PML(229-360)	BamH1-229-PML-Sense	ATTAGGATCCATGATCAGCGCAGAG	PML-360-EcoRV-antisense	TATAGATATCTTTGTCGCAGGCGGCAGA
pcDNA5/FRT/TO-HA-PML	BamH1-PML-Sense	CTATGGATCCATGGTCGAGCCTG	PML-EcoRV-antisense	CCGCGGATATCTAATTAGAAAGGGG
pET28a_SUMO (Smt3p)-PML-Full length	PML-Full-O/L-PetSUMO-F	CTCACAGAGAACAGATTGGTGGATCC GAGCCTGCACCCGCCCGATC	PML-Full-O/L-PetSUMO-R	AGTGGTGGTGGTGGTGGTGCTCGAG CTAAATTAGAAAGGGGTGG
pET28a_SUMO (Smt3p)-3XSUMO2-PML-Full length	3XSUMO2-O/L-PetSUMO-F	ctcacagagaacagattggtggatcc GCCGACGAAAAGCCCAAG	3XSUMO2-O/L-PML-R	ctcggagatcgggcgggtgcaggctc ACCTCCCGTCTGCTGTTGGA
pMAL-c2x-Mad1-full length	Mad1-1-718-O/L-MBP-F	CGAGGGAAGGATTTCAGAATTCGGATCC gaagacctgggggaaaacac	Mad1-1-718-O/L-MBP-R	TAAAACGACGGCCAGTGCCAAGCTT CTACGCCACGGTCTGGCGGCTG
pMAL-c2x-Mad1-596-718 truncation	Mad1-596-718-O/L-MBP-F	CGAGGGAAGGATTTCAGAATTCGGATCC TCGTCCAAGGAGGTGGCAG	Mad1-1-718-O/L-MBP-R	TAAAACGACGGCCAGTGCCAAGCTT CTACGCCACGGTCTGGCGGCTG
pRetroCMV_TO_Human-Mad1-3XFLAG-PuroR	p5-OLP-cmv2-retro-FOR	C CTC CGG ACT CTA GCG TTT AAA CTT AAG CTT GGT ACC GAG CT	p5-OLP-cmv2-retro-REV	TACCCGGTAGAATTCTAGA**CTCGAG**CAGCGGGTTTAAACG
pRetroCMV_TO_Mad1(SIM mutant)-3xFLAG	p5-OLP-cmv2-retro-FOR	C CTC CGG ACT CTA GCG TTT AAA CTT AAG CTT GGT ACC GAG CT	p5-OLP-cmv2-retro-REV	TACCCGGTAGAATTCTAGACTCGAGCAGCGGGTTTAAACG
pcDNA5/FRT/TO-NeonGreen	Xho 1-YFP-Sense	TCAACTCGAGATGGTGAGCAAGGGC	Apa1-YFP-Anti-sense	TTGGGCCCCTACTTGTACAGCTCGTC
pcDNA5/FRT/TO-Mad1-NeonGreen	BamH1-Mad1-sense	CCTGGATCCACCATGGAAGACCTG	EcoR V-Mad1-Anti	TTCTAGATATCTCGTCGCCACGGTCTG
pENTR1A-NeonGreen	p5-Promoter/PA-O/L-pENTRA-FOR	GTCGACTGGATCCGGTACCGAATTC TCTCCCGATCCCCTATGGTG	p5-Promoter/PA-O/L-pENTRA-REV	ATATCTCGAGTGCGGCCGCGAATTC GCTGGTTCTTTCCGCCTCAG
pENTR1A-Mad1-NeonGreen	p5-Promoter/PA-O/L-pENTRA-FOR	GTCGACTGGATCCGGTACCGAATTC TCTCCCGATCCCCTATGGTG	p5-Promoter/PA-O/L-pENTRA-REV	ATATCTCGAGTGCGGCCGCGAATTC GCTGGTTCTTTCCGCCTCAG
pAd/PL-DEST-NeonGreen	none	none	none	none
pAd/PL-DEST-Mad1-NeonGreen	none	none	none	none
GFP-Nucleolin(addgene (Plasmid #28176))	none	none	none	none
pENTR1A-GFP-Nucleolin	EGFP-C1-CMV-O/L-pENTR1A-FOR	GACTGGATCCGGTACCGAATTC AGCCCATATATGGAGTTC	EGFP-C1-PolyA-O/L-pENTR1A-REV	ATCTCGAGTGCGGCCGCGAATTC CGCTTACAATTTACGCGT
pAd/PL-DEST-GFP-Nucleolin	none	none	none	none
pcDNA5/FRT/TO-Mad1(no tag)	BamH1-Mad1-sense	CCTGGATCCACCATGGAAGACCTG	EcoR V-Mad1-Anti	TTCTAGATATCTCGTCGCCACGGTCTG
pcDNA5/FRT/TO-Mad1(1-596,no tag)	BamH1-Mad1-sense	CCTGGATCCACCATGGAAGACCTG	Mad1-596-anti-EcoRV	ATCCTGATATCATGGCAGACTCGCG
pcDNA5/FRT/TO-	BamH1-PML-Sense	CTATGGATCCATGGTCGAGCCTG	PML-EcoRV-antisense	CCGCGGATATCTAATTAGAAAGGGG

3xFLAG-PML				
pcDNA5/FRT/TO-3xFLAG-hMDM2	BamH1-hMDM2-Sense	TTAGGATCCATGGTGAGGAGCAGGCAA	hMDM2-Xho1-Anti	CAGCCTCGAGCGGGAAATAAGTTAGCA
pcDNA5/FRT/TO-2xHA-hMDM2	BamH1-hMDM2-Sense	TTAGGATCCATGGTGAGGAGCAGGCAA	hMDM2-Xho1-Anti	CAGCCTCGAGCGGGAAATAAGTTAGCA
pcDNA5/FRT/TO-Mad1-YFP	BamH1-Mad1-sense	CCTGGATCCACCATGGAAGACCTG	EcoR V-Mad1-Anti	TTCTAGATATCTCGTCGCCACGGTCTG
pcDNA5/FRT/TO-Mad1(SIM mutant)-YFP	BamH1-Mad1-sense	CCTGGATCCACCATGGAAGACCTG	EcoR V-Mad1-Anti	TTCTAGATATCTCGTCGCCACGGTCTG
pRetroCMV_TO_Human-Mad1(SIM mutant)-YFP-PuroR	p5-OLP-cmv2-retro-FOR	C CTC CGG ACT CTA GCG TTT AAA CTT AAG CTT GGT ACC GAG CT	p5-OLP-cmv2-retro-REV	TACCCGGTAGAATTCTAGA**CTCGAG**CAGCGGGTTTAAACG
